# Data-driven hypothesis discovery from disease trajectories in multiple sclerosis

**DOI:** 10.3389/fimmu.2026.1758416

**Published:** 2026-04-14

**Authors:** Niels Jodts, Lorin Werthen-Brabants, Sofie Aerts, Liesbet M. Peeters, Bart Van Wijmeersch, Charlotte Herzeel, Christel Meertens, Roel Wuyts, Tom Dhaene, Dirk Deschrijver

**Affiliations:** 1 IDLab, Universiteit Gent - Imec, Ghent, Belgium; 2 Universitair MS Centrum (UMSC) Hasselt-Pelt, Pelt, Belgium; 3 Biomedisch Onderzoeksinstituut (BIOMED), Universiteit Hasselt, Diepenbeek, Belgium; 4 Rehabilitation Research Center, Universiteit Hasselt, Diepenbeek, Belgium; 5 Revalidatie en MS, Noorderhart, Pelt, Belgium; 6 Data Science Institute (DSI), Universiteit Hasselt, Diepenbeek, Belgium; 7 AI & Algorithms (AIA) Department, Imec, Leuven, Belgium; 8 Declarative Language and Artificial Intelligence (DTAI), KU Leuven, Leuven, Belgium

**Keywords:** clustering, data-driven, disease progression analysis, disease trajectories, hypothesis discovery, multiple sclerosis, real-world cohort, longitudinal data analysis

## Abstract

**Introduction:**

Multiple sclerosis (MS) is an incurable autoimmune disease marked by heterogeneous progression and a lack of reliable biomarkers, complicating prognosis and individualized care. This study introduces a novel trajectory-based statistical approach designed to identify patterns in patient histories within MS populations.

**Methods:**

Using longitudinal clinical data from a real-world cohort of 1,025 MS patients (median follow-up: 6.75 years), two complementary analyses were conducted based on patient trajectory analysis. In the first analysis, the technique is applied to the complete dataset after removal of missing values (n = 985; 11,048 events) to uncover latent progressive trajectories. The second analysis evaluated the techniques’ performance on a smaller, limited-sample cohort (n = 83; 282 events).

**Results:**

Across both analyses, the approach revealed previously unrecognized progression patterns, giving rise to new hypotheses, including an effect of Alemtuzumab on the bowel/bladder function (p*<*0.01, RR = 2.83) and glatiramer acetate on the occurrence of relapses (p*<*0.01, RR = 1.49). Known associations were also confirmed, such as the relationship between relapse activity and brain lesions (p*<*0.01, RR = 1.20).

**Discussion:**

The results demonstrate the method’s robustness across varying dataset sizes, highlight its methodological limitations, and show its potential to uncover previously unseen relationships among MS-specific diagnostic events. These findings provide a foundation for generating novel hypotheses relevant to biomarker discovery and therapeutic optimization.

## 1 Introduction: understanding disease progression in multiple sclerosis

Multiple sclerosis (MS) is an incurable autoimmune disease, characterized by immune-mediated inflammation and neurodegeneration in which the immune system damages the myelin sheath surrounding nerve fibers, leading to progressive neurological disability (1, 2). The expression of MS is categorized according to four distinct clinical courses: clinically isolated syndrome (CIS), relapsing-remitting MS (RRMS), primary-progressive MS (PPMS) and secondary-progressive MS (SPMS), based on the observed pathology of the patient. The clinical courses are considered dynamic and can therefore change over time (3).

Although several disease-modifying therapies (DMTs) exist to slow disease progression and improve patient quality of life, there is currently no cure for MS (1). As the condition advances, clinicians face complex decisions about treatment strategies and long-term care planning. The difficulty lies in the complicated nature of MS prognosis, which arises from the scarcity of viable biomarkers (4), disease progression heterogeneity (2), and the lack of robust predictive tools in clinical practice (5).

In response to the persistent challenges of understanding MS progression, the scientific community has increasingly turned to data-driven strategies (6–8). In general, these strategies can be divided into 1) predictive approaches where future disease activity is forecast based on learned patterns, and 2) modeling approaches where patterns observed across patient populations are modeled. While predictive models often achieve high accuracy as black-box models, their limited interpretability and data-hungriness still hinder routine clinical use. However, they are an effective tool to predict upcoming changes in patients’ disease. In contrast, modeling approaches focus on mapping and interpreting the sequence of clinical events over time, offering more insights into disease mechanisms and subtypes that may not emerge from purely predictive methods.

This study adopts a data-driven modeling approach, Patient TRajectory Analysis (PTRA) (9), to investigate disease progression patterns in MS. Trajectory-based approaches have proven effective in uncovering clinically meaningful patterns in other chronic and acute conditions such as sepsis, chronic obstructive pulmonary disease, bladder cancer, and intensive care unit mortality (10). By capturing temporal relationships between clinical events, PTRA provides a basis for generating novel hypotheses. Additionally, by demonstrating the approach’s feasibility on a small, limited dataset, this work establishes a foundation for applying trajectory analysis to studies where sample sizes are a constraint, thereby widening its potential applicability in MS research and beyond (9).

This study proposes the use of Patient Trajectory Analysis (PTRA) (9) to offer two key contributions in MS research:

1. The uncovering of latent progressive trajectories of a single-center, real-world MS cohort of 1,025 patients for exploratory data analysis to serve as a foundation for hypothesis discovery, and.
2. The validation of the PTRA methodology on a small subset of the dataset through reproduction of a previous statistical study on the safety and effectiveness of Cladribine in MS, thereby assessing its suitability for use in limited-sample clinical research scenarios.

## 2 Methods

This section details the retrospective application of the PTRA method (9) to a real-world anonymized MS cohort dataset. It must be explicitly stated that no clinical experiments were set up or conducted to perform these analyses, as the work relies entirely on retrospective data. First, the dataset characteristics were outlined, followed by a detailed description of the PTRA pipeline. Lastly, a detailed description of the set-up of each experiment was described.

### 2.1 Dataset description: a real-world longitudinal MS cohort dataset

The retrospective dataset contained clinical, magnetic resonance imaging (MRI), treatment, and relapse data from a single-center, real-world MS cohort at the tertiary MS treatment center Noorderhart in Pelt, Belgium. Past clinical data were extracted from the MS data entry portal iMed (iMed, ^©^ 2022 MSBase Foundation, Australia) and included the following features: age, gender, MS course and Expanded Disability Status Scale Scores (EDSS) which follows the definition from Neurostatus (11), an adaptation of the original EDSS scale defined by Kurtzke (12).

A total of 1,025 people with MS were selected for this analysis. After removal of patients with only one recording in the database, a total of 985 patients remained (69.75% female, median age of 43.1 years at baseline, median age of 36.0 years at diagnosis). The median follow-up duration within the dataset was 80.8 months per patient (approximately 6.75 years), while the median number of visits was 15. The EDSS interquartile range was 2.0−4.5 at baseline and 2.0−6.0 at the end of follow-up. Lastly, MS courses were recorded during every visit. The automatic registration of Clinically Isolated Syndrome (CIS) introduced a classification bias. Patients under evaluation who did not meet the diagnostic criteria of MS immediately were automatically encoded as CIS at entry. This protocol inevitably captures patients awaiting specialist confirmation who effectively had RRMS from the start. Therefore, it is impossible to distinguish between patients who genuinely converted from CIS to RRMS within a short interval (e.g., one month) and those who were administratively misclassified at baseline. This limitation leads to an overrepresentation of CIS in the initial dataset. At baseline, CIS appears for 84.97% of patients. By the end of the study, 64.57% had a recorded diagnosis of RRMS, while 19.90% were diagnosed with SPMS. An overview of all demographic data can be found in **Table 1**.

**Table 1:** Demographics of the full MS cohort after removal of patients with only one entry in the database.

Characteristic	Value (n = 985)
Gender (Female), n (%)	687 (69.75%)
Age at diagnosis (years), med [Q1-Q3]^a^	36.0 [28.1 - 45.3]
Age at baseline (first visit, years), med [Q1-Q3]^a^	43.1 [34.3 - 52.9]
Age at end (last visit, years), med [Q1-Q3]^a^	51.3 [41.3 - 61.0]
EDSS at baseline (first visit), med [Q1-Q3]	3.0 [2.0 - 4.5]
EDSS at end (last visit), med [Q1-Q3]	3.0 [2.0 - 6.0]
Total number of visits, med [Q1-Q3]	15 [7.0 - 24.0]
Total follow-up duration (months), med [Q1-Q3]^b^	80.8 [40.4 - 134.8]
MS course at baseline (first visit), n (%)^c^	
CIS	837 (84.97%)
RRMS	0 (0.00%)
SPMS	0 (0.00%)
PPMS	86 (8.73%)
MS course at end (last visit), n (%)^c^	
CIS	5 (0.51%)
RRMS	636 (64.57%)
SPMS	196 (19.90%)
PPMS	86 (8.73%)

^a^ n = 945, missingness: 4.06%.

^b^ n = 939, missingness: 4.67%.

^c^ n = 923, missingness: 6.29%.

The full dataset, which included relapse, MRI, EDSS, treatment and demographic data, was used in the first exploratory analysis, which aimed to assess patient disease trajectories. In the second analysis, a subset of the data was used to test the applicability of the PTRA method on a smaller dataset. This was achieved by comparing results with those of a previous analysis by Aerts et al. (13), which investigated the safety and effectiveness of Cladribine for MS patients who were diagnosed with RRMS. The methodology implemented by Aerts et al. (13) is further elaborated in Section 2.4.

### 2.2 PTRA analytical pipeline

A variety of approaches exist to examine patient trajectories, but they generally follow a similar analytical framework (10). The goal is to extract specific events (A, B, C, D, E,…) from retrospective patient data, identify chronological patterns, and visualize them for analysis. It is important that the selected events are well defined to enable replication and comparison of results. Consequently, diagnoses are often used as events, because they can be defined according to standardized ontologies such as the International Classification of Diseases, 10th Revision (ICD-10) (10). However, context-specific events such as medication changes or observed disease progression indicated by changes in the EDSS or functional system scores (FSS) defined by Kurtzke (12) may be considered.

The analytical pipeline used in this study follows the methodology of Herzeel et al. (9) and proceeds as follows:

1. Definition of trajectory events.
  For every analysis, a set of context-specific events was defined (Event A, Event B, Event C) as discussed in section 2.3 and 2.4.
2. Creation of event pairs.
  All possible ordered pairs were generated from the defined events (*A* → *B*, *A* → *C*, *B* → *C*).
3. Calculation of the relative risk.
  For each pair, the relative risk (RR) of the pair (*A* → *B*) and its reverse (*B* → *A*) was calculated to quantify how much more likely event B is to occur after event A, and vice versa. By definition, an increase in RR indicates an increased likelihood of event B occurring after event A. With a RR of 2 indicating that an event B is twice as likely to occur after event A, compared to B occurring without event A preceding B. The RR is defined as the ratio of:
  • Outcome rate of exposed patients:
  (1) $$OR_{\begin{array}{c} \text{exposed} \end{array}}=\frac{pAB}{pAB+pA\bar{B}}$$
  To calculate the outcome rate of exposed patients shown in Equation 1, all patients exposed to event A were selected. Of these, pAB were patients who experienced event B within a predefined time-range (e.g. [0–1] years) after A, and $pA\bar{B}$ were patients who did not experience B within the defined time-range.
  • Outcome rate of unexposed patients:
  (2) $$OR_{\begin{array}{c} \text{unexposed} \end{array}}=\frac{p\bar{A}B}{p\bar{A}B+p\bar{A}\bar{B}}$$
  To calculate the outcome rate of unexposed patients shown in Equation 2, an equal number of patients not exposed to A were randomly selected, matched for age and sex. Within this group, $p\bar{A}\bar{B}$ represents patients who experienced event B despite not being exposed to A within the time-range, and $p\bar{A}\bar{B}$ represents patients who experienced neither A nor B.
  The relative risk is calculated as (Equation 3):
  (3) $$RR=\frac{OR_{\text{exposed}}}{\begin{array}{c} OR_{\text{unexposed}} \end{array}}$$
4. Filter non-significant event pairs.
  To account for variability arising from the random selection of unexposed patients during RR (RR) calculation, the procedure was repeated 10,000 times to derive an empirical p-value. Event pairs were retained as significant only if they exhibited a *RR >* 1 and a *p <* 0.01 (9).
5. Creation of trajectories.
  In case the RR of both the pair and its reverse exceeded 1, a binomial experiment was used to select the more prominent direction within the data. Based on a chosen minimum patient count and time-ranges, the event pairs were chained into trajectories. The time-range filters for short-term or long-term patterns, while the minimum patient count prevents visualization clutter from rare trajectories.
6. Visualization.
  The Markov Clustering Algorithm was used to generate a clear, clustered overview of the trajectory data (14).
7. Calculate cluster statistics.
  Lastly, for every cluster, the patient population was examined. Mean and standard deviation of patient age at the first recorded event was determined. Patient count and gender proportions were determined for further comparison to the original patient population.

The PTRA method allows for different perspectives of a longitudinal dataset by changing the allowed time between events, the number of patients per trajectory and the definition of context-specific events. In order to find novel hypotheses in the dataset, it is advisable to use different patient counts and time-ranges to create trajectories at different levels of detail. In the next sections, the dataset selection, context-specific events and settings for the analytical pipeline were detailed for the two analyses.

For all clusters with more than two events, the cluster statistics of the patient subset were compared to the full patient population. The full patient cohort was treated as a finite population of which a subset has been sampled. The gender distribution was compared to the total population using Python (version 3.12) and the SciPy library. Comparison was done through a Z-test for proportions with statistical significance set at *p*< 0.05.

### 2.3 Analysis 1: uncovering the disease trajectories in a small, real-world MS cohort

For this first analysis the full dataset of 985 MS patients was used. The purpose of the first analysis was to explore the MS cohort dataset and uncover previously unseen patterns in the patient disease trajectories. To achieve this, the chosen diagnostic events intentionally deviated from conventional trajectory modeling approaches by using context-specific self-defined events rather than standardized ICD-10 diagnostic codes. This choice enabled insights tailored to MS trajectories, offering a deeper understanding of the progression patterns.

Diagnostic events are classified using a two-tier hierarchy: a broader main category and a specific subcategory beneath it. To prevent data overlap, each subcategory belongs to exactly one main category. If an event lacks a distinct subcategory, the main category acts as both. The definitions for the main and subcategories are:

1. MS course change: a recorded change in MS course indicating disease progression (3). The date of classification was used as event date. The subcategories are: change to CIS (at first recording), change to RRMS and change to SPMS.

2. Relapse: Defined as a sudden onset or worsening of neurological symptoms that persists for at least 24 hours. The date of onset was used as event date. There were no subcategories for relapse events.

3. MRI activity: A recorded increase in the number of lesions on T2-weighted and Short Tau Inversion Recovery (STIR) images, confirmed via manual counting and review by a neurologist. This manual counting was performed following the automated detection of an increase in overall T2 lesion volume. The date of recording was chosen as event date. The subcategories signified the region with lesion activity: the spinal cord region (MRI SP) or the brain region (MRI BR).

4. +/- EDSS: A persistent change in EDSS, confirmed by one subsequent visit. An increase in EDSS (+EDSS) is indicative of disease worsening, whereas a decrease (-EDSS) is indicative of improvement (12). The date with the first recorded change was used as event date. There were no subcategories for EDSS changes.

5. +/- FSS: A persistent change in FSS confirmed by one subsequent visit. An increase in FSS represented worsening of the condition, while a decrease in score meant an improvement of the condition. When multiple FSS increased or decreased between visits, they were merged into one event: “+/-FSS”. The subcategories are based on the seven FSS and the ambulation score defined by Kappos et al. (11), which was adapted from Kurtzke (12): +/-bowel/bladder; +/-pyramidal; +/-cerebellar; +/-brainstem; +/-mental; +/-sensory, +/-visual and +/-ambulation The date with the first recorded change was used as event date.

6. DMT change: A change in disease modifying treatment. Changes in dosage or frequency were not recorded. All DMTs are grouped together based on mode of action and strength. The DMTs, their group, abbreviation and an explanation of why they are grouped is further elaborated in **Table 2**. The subcategories are the DMT groups. The first day of a new treatment was recorded as event date.

**Table 2:** List of disease modifying treatments (DMTs) present in the MS cohort dataset.

Abbreviation	Disease modifying treatment	Mode of action
IFN/GLAT	(peg)-Interferon-beta-1a/b, Glatiramer acetate	Immunomodulating DMTs without lymphocyte depletion
CD20	Rituximab, Ocrelizumab, Ofatumumab	Monoclonal antibodies targeted towards CD20-antigen, causing B-cell depletion
OTH	Mitoxantrone, Methotrexate, Azathioprine, Cyclophosphamide	Non-selective suppression of the immune system
TRF	Teriflunomide	Pyrimidine synthesis inhibitor
DMF	Dimethyl Fumarate	Nrf2 pathway activator/immunomodulator
FNG	Fingolimod	Sphingosine 1-phosphate (S1P) receptor modulator
NTZ	Natalizumab	*α*4-integrin receptor antagonist
ALZ	Alemtuzumab	Anti-CD52 monoclonal antibody

All DMTs are grouped according to their primary mode of action and strength. Similar treatments are grouped together, while treatments with distinct mechanisms are listed individually.

**Figure 1:**
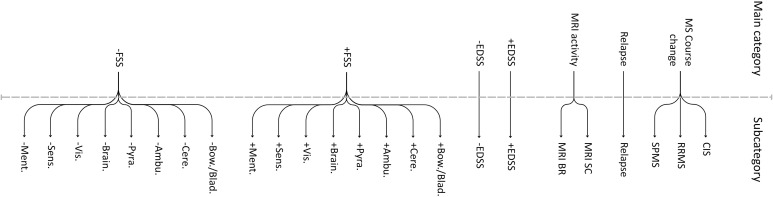
Tree diagram of the context-specific diagnostics events that were defined for the first analysis. The events are structured hierarchically, with the main category at the top and the corresponding subcategories at the bottom. Every diagnostic event has a definition and contains at least one subcategory. MS Course change: a recorded change in MS course indicating disease progression. The subcategories are: change to CIS (at first recording), change to RRMS and change to SPMS. Relapse: a sudden onset or worsening of neurological symptoms that persists for at least 24 hours. MRI activity: An MRI recorded increase in the number of lesions (from T2 and STIR-weighted MRI images). Subcategories: region of lesion activity: spinal cord region (MRI SC) or brain region (MRI BR). +/- EDSS: A persistent change in EDSS, confirmed by one subsequent visit. +/-FSS: A persistent change in FSS confirmed by one subsequent visit. The subcategories are based on the seven FSS and the ambulation score defined by Kappos et al. (11), which was adapted from Kurtzke (12): bowel/bladder; pyramidal; cerebellar; brainstem; mental; sensory, visual and ambulation.

A visual overview of the main- and subcategories is given in **Figure 1**. Missing data on MS course changes as mentioned in **Table 1** could not be added as diagnostic events. This could lead to an under-representation of MS course change events. It is therefore important to consider that the full impact of course changes cannot be retrieved from this analysis.

The exploratory analysis of the full MS dataset was performed with six different set-ups: three separate time-ranges were defined: [0–1 years], [0–2 years], and [1–5 years]. The first two time-ranges allow for a visual overview of short-term event sequences, while the last time-range allows for a long-term view of the disease where the time between events is up to five years and short-term event pairs that appear within one year are excluded. For each of these three time-ranges an experiment is performed on the main categories and on the subcategories. Resulting in six total experiments. The minimum number of patients present in one trajectory was set to 10, approximately 1% of the total patient count. This was chosen to ensure that most of the significant trajectories are shown, whereas rare events are ignored because these are unlikely to be statistically valid and they clutter the output. The data is explored visually and compared to literature.

### 2.4 Analysis 2: Validation of patient trajectories for limited-size datasets

In the second analysis, only a subset of the MS cohort data was used to validate the findings of the PTRA pipeline for limited-sized datasets by attempting to reproduce the findings of Aerts et al. (13) on the same dataset. To achieve this subset of the MS cohort, the following inclusion criteria were used: 1) only patients diagnosed with relapsing-remitting MS (RRMS), 2) who initiated Cladribine (CLAD) treatment between August 2018 and November 2021, and 3) had sufficient follow-up data to allow for a CLAD effectiveness evaluation. This meant patients must have had completed at least one CLAD treatment cycle (two weeks within the first year of treatment). Lastly, a database lock was set at the 8th of April, 2022.

A total of 83 patients were eligible for this selection, with a limited number of patients excluded from the original cladribine dataset as they did not meet the above specified requirements. The baseline EDSS was determined as the closest EDSS recorded within a range of six months before CLAD initiation or three months after. An overview of the relevant demographic data is shown in **Table 3**.

**Table 3:** Demographics of the MS cohort subset.

Characteristic	Value (n=83)
Gender (Female), n (%)	53 (63.86%)
Age at cladribine start, med [Q1-Q3]	43 [33.5 - 51.0]
Years since diagnosis, med [Q1-Q3]	6 [2.0 - 11.0]
Total follow-up duration (months), mean [std] (range)	21.7 [10.3] (4.0 - 35.0)
EDSS at baseline (first visit), med [Q1-Q3]	2.5 [1.5 - 3.75]
EDSS at end (last visit), med [Q1-Q3]	3.0 [2.0 - 6.0]*

*n = 82.

The method aimed at reproducing the following findings from the study of Aerts et al. (13), the amounts are put between brackets:

1. The number of patients who experienced at least one sign of evidence of disease activity (EDA) during the full study (n=20).
2. The count of relapse (n=21) and confirmed disability worsening (CDW) (n=6) events during the full study. The number of MRI events were not counted in the study, only the number of new or enlarging brain MRI lesions (n=11).
3. The number of patients with MRI (n=5), relapse (n=14), or CDW (n=6) events during the full study.
4. A trend of earlier disease activity in patients who switched from FNG to CLAD.
5. A trend of earlier disease activity in patients who received two or more different DMTs prior to CLAD initiation.
6. Comparison between patients who received fewer than two DMTs prior to CLAD initiation compared to those who received two or more: a significant difference in duration until CDW during the study (borderline non-significant, p = 0.053). All other comparisons between patients groups or the influence of DMT switches to CLAD were found to be non-significant.

For the purpose of partially reproducing the findings of Aerts et al. (13), the following diagnostic events were defined:

1. EDA-3: evidence of disease activity determined by one of three indications. The subcategories of EDA-3 are:  • Relapse: Defined as a sudden onset or worsening of neurological symptoms that persists for at least 24 hours and being ≥ 30 days apart from any infection or fever. The date of onset was used as event date.  • CDW: confirmed disability worsening. Any increase in EDSS that was sustained for ≥ 6 months. The first date of recorded increase was chosen as event date.  • MRI activity: Recorded increase in the number of lesions on MRI images from T2 and STIR weighted images based on manual counting and review of a neurologist. Manual counting happened after detecting an increase of T2 lesion volume. The date of recording was chosen as event date.2. NEDA-3: The absence of EDA-3 within the full follow-up duration of the patient. When a patient has no recorded events, NEDA-3 is added as main- and subcategory. The event date was set at the following date: first recorded date + time-range of the study. This ensured that the event would always be at the end when performing PTRA.3. Prior DMT: the last DMT treatment received before CLAD initiation. The categories of the prior DMTs are shown in **Table 2** with the same categorization as for the previous analysis.4. Prior DMT count: the number of different treatments a patient has received before CLAD initiation. The two categories are: two or more DMTs (≥2 DMTs) or less than two DMTs (*<* 2 DMTs). The date of CLAD initiation was chosen as event date.

The PTRA pipeline counts all event occurrences when changing the requirements for the RR score and p-value to 0 and 1 respectively. This creates a graph with all trajectories present in the dataset allowing to verify the first three findings. To detect trends of early disease activity after CLAD initiation, three time-ranges were chosen. The first only considered all events that were within one year of CLAD initiation. The second experiment considered all events within two years of initiation and the final experiment considered all recorded events during the study. To test trends of earlier activity, a statistically significant increase in RR score for the short-term experiments was expected. Due to the low number of patients in the dataset the minimum patient count was set to 1.

## 3 Results: disease trajectories in MS

Before presenting the results, it is important to clarify how the resulting trajectories should be interpreted. By definition, an increase in RR indicates an increased likelihood of event B occurring after event A. This measure is based solely on observed data and does not imply causality. Moreover, the data collection protocol employed by the source database does not guarantee the time-synchronized testing of events A and B. Event B could occur before A but still be recorded as following A due to infrequent measurements. Nevertheless, observing an increased RR offers insight into the interrelationships between events and can help generate new hypotheses for further investigation.

### 3.1 Analysis 1: MS disease trajectories in a real-world MS cohort

The PTRA pipeline was applied to 11,048 events derived from 985 patients. By clustering event pairs derived from patient history that show a statistically significant increase in RR (*RR >* 1; *p <* 0.01), a visual overview of the dataset is established. To find previously documented patterns and identify novel hypotheses regarding MS progression, the analysis was conducted using three specific time-ranges: [0–1 years], [0–2 years], and a long-term time-range of [1–5 years]. These ranges help to distinguish between immediate and gradual disease activity.

**Figure 2:**
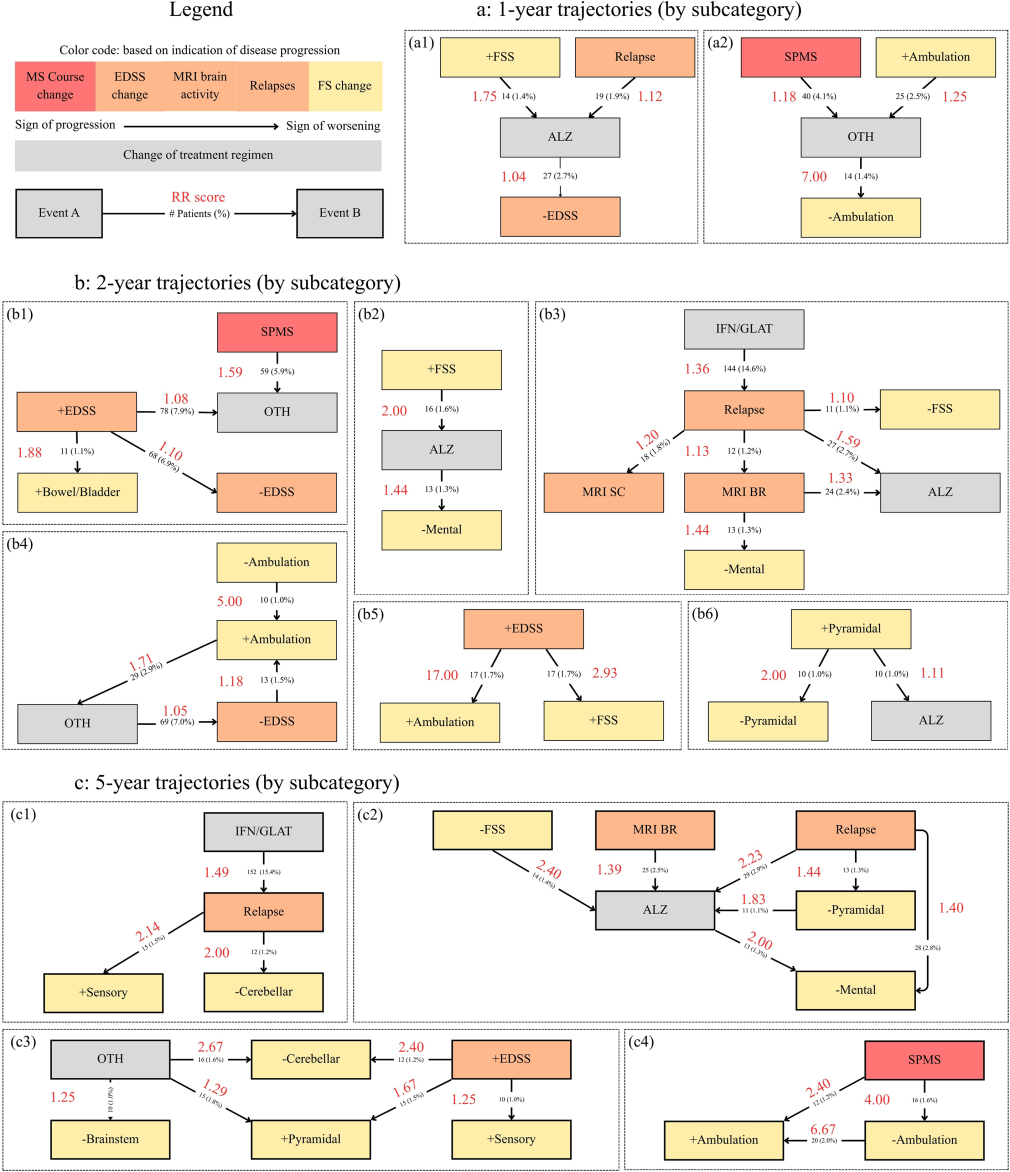
Patient trajectories found in the full MS cohort (n=985). Nodes are context-specific diagnostic events by subcategory. The events are color coded based on the impact on disease worsening they represent. Red indicates changes in disease course, reflecting significant worsening. Orange denotes disease activity, while yellow represents changes in functional system scores. Lastly, treatment changes are shown in gray. Edges are event pairs (*A* → *B*) that have a statistically significant (*p <* 0.01) increase in relative risk (RR) score. By definition, an increase in RR indicates an elevated likelihood of event *B* occurring after event *A*. A score of 2 indicates event *B* is twice as likely to occur after event *A*, compared to event *B* occurring without event *A* preceding event *B*. Edges are accompanied by absolute and relative (between brackets) patient count and RR score in red. **(A)** Disease trajectories where diagnostic events happen sequentially within one year. **(B)** Disease trajectories where diagnostic events happen sequentially within two years (*RR* ≥1.05). **(C)** Disease trajectories where diagnostic events happen sequentially after one year, with at most five years of temporal separation (*RR* ≥1.25). Abbreviations: SPMS, change to secondary progressive disease course; MRI SC, MRI recorded activity in the spinal cord region; MRI BR, MRI recorded activity in the brain region; +/-EDSS: increase/decrease in EDSS score confirmed by one subsequent visit; +/- FSS, increase/decrease of multiple FSS confirmed by one subsequent visit; IFN/GLAT, change to (peg)-Interferon-beta-1a/b, Glatiramer acetate; OTH, change to Mitoxantrone, Methotrexate, Azathioprine, Cyclophosphamide.

In **Figure 2**, all statistically significant clusters (*p <* 0.01) are displayed in directed graphs. Every graph node signifies a diagnostic event, while every edge is accompanied by the absolute and relative (between brackets) patient count, as well as the RR between event A and event B. These graphs only show “large” clusters with at least three diagnostic events and each cluster has an identifier (a1, a2, b1, b2…). The demographic data of patients per cluster is shown in **Table 4**. The remaining clusters with only two events (one event pair) are shown in their respective tables.

**Table 4:** Demographic data on the clusters visible in **Figure 2**.

Cluster ID	Mean age (sd)	Female (%)	n (%)
Figure 2a			
(a1)	42.30 (9.15)	42 (70.00)	60 (6.09)
(a2)	48.90 (9.49)	56 (70.89)	79 (8.02)
Figure 2b			
(b1)	48.72 (10.69)	**148 (64.07)**	231 (23.45)
(b2)	45.14 (8.28)	20 (68.97)	29 (2.94)
(b3)	39.03 (10.80)	110 (75.34)	146 (14.82)
(b4)	51.09 (10.78)	80 (66.12)	121 (12.28)
(b5)	51.62 (9.91)	27 (79.41)	34 (3.45)
(b6)	44.90 (10.62)	15 (75.00)	20 (2.03)
Figure 2c			
(c1)	41.93 (11.14)	**131 (80.37)**	163 (16.55)
(c2)	41.82 (9.38)	218 (73.65)	296 (30.05)
(c3)	50.95 (11.79)	225 (70.75)	318 (32.28)
(c4)	55.12 (11.08)	24 (75.00)	32 (3.25)

Age is determined at the time of the first event where the patient is present in the cluster. Relative percentages are determined based on the total number of patients in the cohort (n=985). Gender proportions that differ significantly from the full patient cohort are highlighted (*p <* 0.05, one-sample Z-test for proportions, reference distribution: 69.75% female).

#### 3.1.1 1-year trajectories

For the [0–1 year] short-term time-range, only a limited number of statistically significant trajectories are observed. Two large clusters are found as shown in **Figure 2a**. Cluster-specific information on the demographics of cluster a1 and a2 are shown in **Table 4**.

The first pattern that appeared over both clusters is the following: a measured disease worsening in the form of 1). an increase in FSS (+Mental, +Ambulation) 2), a worsening of the disease course (to SPMS), or 3), the appearance of a relapse; followed by a change of treatment; after which an improvement of the patients’ condition is observed. In more detail, worsening followed by a change in treatment is shown in the following trajectories: “+*FSS* → *ALZ*”, “*Relapse* → *ALZ*” and “+*Ambulation* → *OTH*”, “*SPMS* → *OTH*”. The trajectory “*SPMS* → *OTH*” stood out in patient count with 40 patients or 4.1% of the total cohort (*RR* = 1.18). Improvements after a DMT change appear as “*OTH* →−*Ambulation*” with a large RR of 7.00 and “*ALZ* →−*EDSS*” with *RR* = 1.04.

Lastly, two more trajectories were found significant, but only had a single event and are therefore not shown in the clustered overview: “+*Mental* →−*Mental*” (*RR* = 2.38, *n* = 19) and “*MRI BR* → *MRI SC*” (*RR* = 1.20, *n* = 12). No statistically significant difference is found between the gender distribution in the clusters compared to the full MS cohort and no trajectories are found when using only the main categories.

#### 3.1.2 2-year trajectories

Analogous to the 1-year trajectories, all clustered trajectories within a time-range of [0–2 years] having at least three events are shown in subfigure 2b. All other trajectories with *RR* ≥ 1.25 are displayed in **Table 5** and the cluster demographics are provided in **Table 4**.

**Table 5:** All 2-year trajectories that consist of only two events per cluster, sorted by relative risk (RR) score with *RR* ≥ 1.25.

Cluster	Patient (%)	RR	Cluster	Patient (%)	RR
+ Mental → - Mental	33 (3.35%)	4.71	+EDSS → ALZ	33 (3.35%)	1.74
+FSS → + Ambulation	10 (1.02%)	3.33	+FSS → - Mental	11 (1.12%)	1.57
+ Mental → + Ambulation	12 (1.22%)	2.40	-FSS → + Mental	10 (1.02%)	1.25
+EDSS → + Pyramidal	19 (1.93%)	2.38			

These trajectories show a statistically significant increase in RR score (*p <* 0.01). Absolute and relative patient count are shown (n=985). Trajectories where *RR <* 1.25 can be found in the **Supplementary Material**.

Similar trajectories found in the 1-year trajectories reappear in the 2-year trajectories, albeit with an increase in RR and patient counts. When comparing the results, the largest increase in RR in **Figure 2** happens between the “*Relapse* → *ALZ*” trajectory of both experiments with ∆*RR* = 0.47. The largest difference recorded in **Table 5** is in the “+*Mental* →−*Mental*” trajectory with an increase in RR from 2.38 in the 1-year trajectories to 4.71 in the 2-year trajectories (∆*RR* = 2.33). Additionally, changes to OTH and ALZ as DMT are still observed to happen within two years of disease worsening: “*SPMS* → *OTH*” (*RR* = 1.59, ∆*RR* = 0.41), “+*FSS* → *ALZ*” (*RR* = 2.00, ∆*RR* = 0.25) and “+*Ambulation* → *OTH*” (*RR* = 1.71, ∆*RR* = 0.46). The “+*EDSS* → *OTH*”, “*MRI BR* → *ALZ*” and “+*Pyramidal* → *ALZ*” trajectories are new event pairs that follow the same pattern. Both the “*ALZ* →−*EDSS*” and “*OTH* →−*Ambulation*” did not remain significant in the 2-year analysis.

Clusters b1 and b5 both show that an increase in EDSS is connected to an increase in other FSS. Examples of this are “+*EDSS* → +*Bowel/Bladder*”, “+*EDSS* → +*Ambulation*” and “+*EDSS* → +*FSS*” with RR of 1.88, 17.00 and 2.93 respectively. In **Table 5** this is observed in “+*FSS* → +*Ambulation*” (*RR* = 3.33) and “+*EDSS* → +*Pyramidal*” (*RR* = 2.38). Fluctuations in FSS appear in cluster b4 and b6 where “−*Ambulation* → +*Ambulation*” and “+*Pyramidal* →−*Pyramidal*” are present with a large RR of 5.00 and 2.00 respectively. The same pattern can be found in **Table 5** as well. Two treatment changes are followed by an improvement of the disease, “*OTH* →−*EDSS*” in cluster b4 (*RR* = 1.05) and “*ALZ* →−*Mental*” in cluster b2 (*RR* = 1.44). These two event pairs are different from the ones observed in the 1-year trajectories but still indicate a change of treatment followed by an improvement of the disease. Lastly, IFN/GLAT is associated with an increased RR for relapse with 144 patients having such an event in their history (cluster b3, *RR* = 1.36).

For cluster b1 the proportion of females in the subset (64.07%) differed significantly from the total population (69.75%; Z-test for proportions: *p* = 0.031), indicating an overrepresentation of males in the first cluster. All other clusters did not differ significantly in gender distribution. Apart from the event pairs in **Figure 2** and **Table 5**, the results on the main categories show two event pairs that are statistically significant: “+*EDSS* → +*FSS*” (*RR* = 1.13) and “−*EDSS* → +*FSS*” (*RR* = 1.02).

#### 3.1.3 5-year trajectories

Lastly, for the 5-year trajectories, all clustered trajectories with at least three events and *RR* ≥ 1.25 are shown in subfigure 2c. The removal of trajectories with a RR below 1.25 helps filter out marginal associations and focus on the most meaningful trajectories, while also removing clutter from the directed graphs. All other clusters with fewer than three events are included in **Table 6** with *RR* ≥ 1.25.

**Table 6:** All 5-year trajectories that consist of only two events per cluster, sorted by relative risk (RR) score with *RR* ≥ 1.25.

Cluster	Patient (%)	RR	Cluster	Patient (%)	RR
OTH → + Ambulation	37 (3.76%)	18.50	- Cerebellar → + Mental	11 (1.12%)	2.20
+FSS → + Ambulation	22 (2.23%)	7.33	Relapse → + Bowel/bladder	15 (1.52%)	2.14
+ Mental → + Ambulation	18 (1.83%)	3.00	- Pyramidal → + Mental	12 (1.22%)	2.00
+ Mental → + Pyramidal	20 (2.03%)	2.86	FNG → ALZ	30 (3.05%)	1.67
ALZ → + Bowel/bladder	17 (1.73%)	2.83	- Mental → + Bowel/bladder	10 (1.02%)	1.67
ALZ → + Ambulation	16 (1.62%)	2.67	SPMS → +FSS	17 (1.73%)	1.55
SPMS → OTH	87 (8.83%)	2.49	-FSS → + Mental	10 (1.02%)	1.43
+ Mental → + Sensory	12 (1.22%)	2.40	IFN/GLAT→+ Bowel/bladder	14 (1.42%)	1.40
OTH → +FSS	29 (2.94%)	2.23	+EDSS → + Bowel/bladder	14 (1.42%)	1.40
-EDSS → + Ambulation	29 (2.94%)	2.23	FNG → + Bowel/bladder	11 (1.12%)	1.38
+FSS → + Mental	20 (2.03%)	2.22			

These trajectories show a statistically significant increase in RR score (*p <* 0.01). Absolute and relative patient count are shown (n=985). Trajectories where *RR <* 1.25 can be found in the **Supplementary Material**.

Similar to previous experiments, IFN/GLAT is still associated with an increased RR for relapse events (*RR* = 1.49) as seen in cluster c1, while the increase in patient count is limited to a relative increase of 5.5% (∆*n* = 8). A change of DMT to ALZ after a relapse or MRI activity remains significant, comparing the relative risk to the 2-year trajectories we find: *RR* = 2.23, ∆*RR* = 0.64 and *RR* = 1.39, ∆*RR* = 0.06. Furthermore, the positive influence of ALZ on the mental FSS (*RR* = 2.00, ∆*RR* = 0.56) also showed an increase in RR when compared to the 2-year trajectories.

The following new observations were made with regards to the FSS: 1), after relapses, improvements in FSS in both cluster c1 and c2 are observed (“*Relapse* →−*Cerebellar*”, “*Relapse* →−*Pyramidal*”); 2), in the results, OTH is shown to decrease the cerebellar and brainstem FSS, but increase the pyramidal and ambulatory FSS as shown in cluster c3 and **Table 6** with “*OTH* → +*Ambulation*” having a high RR of 18.50; 3), ambulatory FSS changes are linked to cluster c4 whose initiating event is the transition to SPMS, showing a high RR for the “*SPMS* →−*Ambulation*” and “*SPMS* → +*Ambulation*” trajectories of 4.00 and 2.40 respectively; and lastly, 4), ALZ is shown to be related to an increase in FSS scores in the trajectory table: “*ALZ* → +*Bowel/Bladder*” and “*ALZ* → +*Ambulation*”. When assessing **Table 6** fully, one can notice how all outcome events indicate worsening of the FSS, with the exception of “*SPMS* → *OTH*” and “*FNG* → *ALZ*”. Both the ambulatory system and the bowel/bladder system appear most often, with all trajectories ending in “+*Ambulation*” having a RR higher than 2.20.

For cluster c1 there is a significant difference in gender distribution of females (80.37%) compared to the full MS cohort (69.75%; Z-test for proportions: *p* = 0.0012), while no significant differences were found for the other clusters. On top of the previous results, the analysis of the main categories shows that the event “−*EDSS*” is paired with “−*FSS*”, “+*EDSS*” and “+*FSS*” with RRs of 1.02, 1.26 and 1.25 respectively. Additionally, both “*Relapse* → +*FSS*” (*n* = 77(7.8%)) and “*Relapse* → *MRI activity*” (*n* = 59(6.0%)) were statistically significant trajectories with a RR of 1.77 and 1.05 respectively.

### 3.2 Analysis 2: MS disease trajectories on a limited dataset

For the reproductive analysis of the CLAD study of Aerts et al. (13) a total of 282 diagnostic events were used from 83 patients. From these diagnostic events, the presence of EDA events in the dataset can be found. A total of 21 relapses, six CDW events and six MRI activity events were recorded from respectively 14, six and five patients. A total of 20 patients had recorded EDA. Patients in the “*<* 2 prior DMTs”-group were found to be significantly younger at the start of CLAD initiation compared to the “≥ 2 prior DMTs”-group (37.27 ± 9.57 vs. 46.11 ± 10.50 years; *p* = 0.0002). The difference in gender distribution was not statistically significant (*p* = 0.4308).

**Figure 3:**
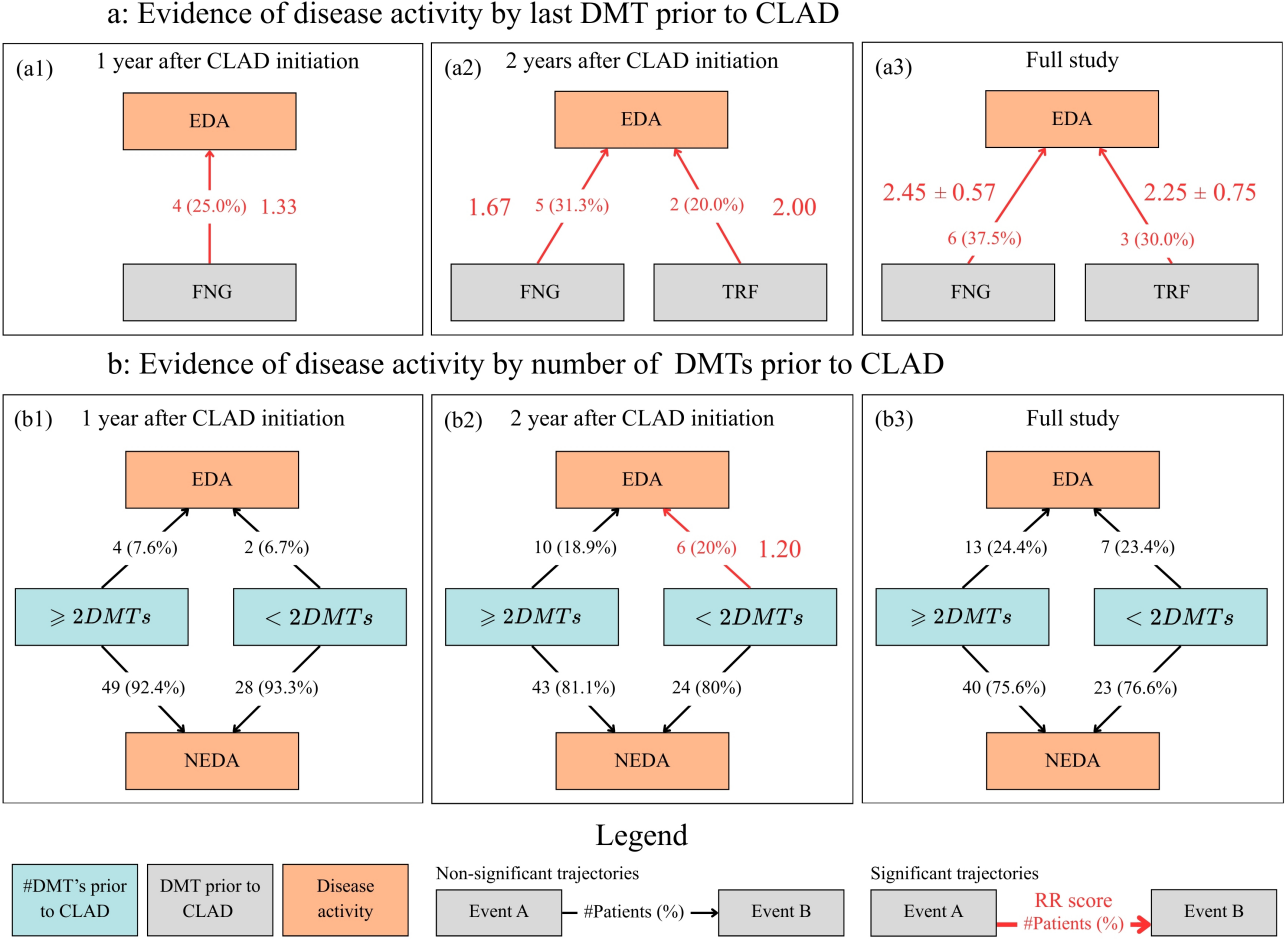
Patient trajectories found in the MS cohort subset (n=83). Nodes are context-specific diagnostic events, while edges show event pairs (*A* → *B*). Pairs that have a statistically significant (*p <* 0.01) increase in relative risk (RR) score are shown in red. Trajectories that are non-significant are shown in black. All edges are accompanied by absolute and relative (in brackets, compared to the number of patients that have *event A* recorded) patient count. Selected events were filtered using the time since start of Cladribine initiation to distinguish short-term response from long-term response. **(A)** Evidence of disease activity based on the last disease modifying treatment received prior to Cladribine initiation. **(B)** Evidence of disease activity based on the number of disease modifying treatments received prior to Cladribine initiation. Abbreviations: DMT, Disease modifying treatment; CLAD, Cladribine; EDA, Evidence of disease activity (recorded MRI activity, confirmed disability worsening or recorded relapses); NEDA, no evidence of disease activity (absence of any recorded EDA within the duration of the study); FNG, Fingolimod as last DMT prior to CLAD initiation; TRF, Teriflunomide as last DMT prior to CLAD initiation; *<* 2*DMTs*: less than two DMTs received prior to CLAD initiation; ≥2*DMTs*: At least two DMTs received prior to CLAD initiation.

The results from the PTRA analysis are shown in **Figure 3** and **Table 7**. **Figure 3a** shows that only FNG and TRF have a significant increase in RR score (*p <* 0.01) with regards to evidence of disease activity, with “*FNG* → *EDA*” already having an RR of 1.33 only one year after CLAD initiation. The RR score for FNG is found to increase with study duration from 1.33 to 2.45(± 0.57), the RR score of TRF also increases from 2.00 to 2.25(± 0.75). In **Table 7** FNG and TRF all show a significant relationship to relapses, with “*FNG* → *Relapse*” being significant for all three durations after CLAD initiation. “*DMF* → *CDW*” appears with a significantly increased RR score of 2.00 for events within two years of CLAD initiation.

**Table 7:** All event pairs with a statistically significant increase in relative risk score (*p <* 0.01) found across the Cladribine (CLAD) study reproduction (13).

	Event pair	#Patient (%)	RR (sd)
1 year after CLAD initiation			
	FNG → Relapse	2 (12.5)	2.00
2 years after CLAD initiation			
	FNG → Relapse	3 (18.8)	1.50
	TRF → Relapse	2 (20.0)	2.00
	FNG → CDW	2 (12.5)	2.00
	DMF → CDW	2 (11.1)	2.00
	*<* 2 DMTs → Relapse	4 (13.3)	1.33
	*<* 2 DMTs → CDW	2 (6.67)	2.00
	*<* 2 DMTs → MRI	2 (6.67)	2.00
Full study duration			
	FNG → Relapse	4 (25.0)	2.60 (1.18)
	TRF → Relapse	3 (30.0)	1.75 (0.85)
	FNG → CDW	2 (12.5)	1.60 (0.49)
	*<* 2 DMTs → Relapse	5 (16.7)	1.17 (0.20)

“*<* 2 DMTs”, less than two different disease modifying treatments received prior to CLAD initiation; CDW, confirmed disability worsening; FNG, last DMT used prior to CLAD initiation is fingolimod; TRF, last DMT used prior to CLAD initiation is Teriflunomide; DMF, last DMT used prior to CLAD initiation is Dimethyl Fumarate. Pairs are ordered per experiment based on the time since CLAD initiation. For the full study duration, the relative risk varied for every run. Therefore, mean and standard deviation for 10 tests are shown. Relative patient count is based on the number of patients that had event A in their patient history.

With regards to the number of prior DMTs received before CLAD initiation, subfigure 3b shows only one trajectory with a significant increase in RR score. The “*<* 2 *DMTs* → *EDA*” event pair has a RR of 1.20 (*p <* 0.01) with six patients experiencing worsening within two years after CLAD initiation. For every type of EDA (MRI activity, CDW and relapse) people who received fewer than two DMTs before CLAD initiation had a significant increase in RR compared to those who received at least two different treatments within two years of CLAD initiation with the RR ranging from 1.33 to 2.00.

## 4 Discussion: what disease trajectories tell us about disease progression

### 4.1 Analysis 1: exploration of MS disease trajectories in a real-world MS cohort

#### 4.1.1 1-year trajectories

The pattern of worsening, followed by a change of treatment and an improvement, which appears in both cluster a1 and a2 of **Figure 2**, can be attributed to the fluctuating nature of RRMS and relapses (1, 15) as well as the efficacy of treatments. The trajectories inform about past treatment decisions based on the progression of the patient. Historically, the management of the transitional phase from RRMS to SPMS relied on broad-spectrum immunosuppression. Mitoxantrone (16) and Cyclophosphamide (17) were established as efficacy standards for slowing ambulatory worsening in patients with active progressive disease, which both belong to the OTH category. This is seen in the trajectories where “+Ambulation” and “SPMS” are both followed by OTH. The increased RR for the “*OTH* →−*Ambulation*” trajectory, shows how this decision frequently leads to improvement of the ambulatory system. Similarly, the data shows that ALZ was often used to treat relapses, which in many instances leads to an overall improvement of the patients’ condition. This observation is further confirmed in previous studies by Moser et al., Herrero-Poch et al. (18, 19). It is however important to note that these results on the influence of DMT changes also represent local treatment procedures, which therefore limit the generalizability of these findings. Lastly, the observed fluctuations of the mental FSS can be attributed to RRMS fluctuations, or to the mental state of patients that go through the progression of MS and the accompanying changes in DMTs. Similarly, the link between “MRI BR” and “MRI SC” can be explained by the appearance of both lesions in the brain and the spinal cord within a short time.

#### 4.1.2 2-year trajectories

The recurrence of trajectories from the previous experiment indicates two things: first, the duration between event A and event B also falls within a 1-to-2-year time-frame; and second, alternative events leading to outcome B do not diminish the statistical significance of the *A* → *B* sequence. ALZ appears multiple times in both clusters of subfigure 2b and **Table 5**, which reaffirms how ALZ was historically used in the MS cohort for the treatment of overall worsening of the patient (“+FSS”, “Relapse”, “+Pyramidal”). The increase in RR for both the “*Relapse* → *ALZ*” and “+*Mental* →−*Mental*” shows how these events are still relevant within a 1-to-2-year time-frame.

Cluster b1 and b5 signify the relationship between the EDSS total score and the FSS, which is a logical observation as this is defined by the make-up of the EDSS scale (12). A higher RR is expected in the changes that more quickly lead to an increase in EDSS total score, like +ambulation and +FSS indicating the worsening of multiple functional systems. The fluctuations in FSS scores that appear in clusters b4 and b6 as well as in b1 (“+*EDSS* →−*EDSS*”) confirm that spontaneous improvement or natural disease fluctuations occur in MS, which has been established in previous studies (1, 5). The increased risk of relapses after a therapy switch to IFN/GLAT could be attributed to other treatments having relapse-reducing capabilities compared to IFN/GLAT, as found by Etta et al. (20), which could explain the increased RR. Additionally, Cofield et al. (21) have confirmed that a switch from NTZ to glatiramer acetate can significantly increase the odds of relapses compared to switching to FNG. Iaffaldano et al. (22) further observed that staying on NTZ reduces the risk of relapses, while a switch to IFN/GLAT can increase the annualized relapse rate (21, 22). However, caution is advised when interpreting the results, as comparing patients on different treatment regimens can lead to incorrect conclusions.

One of the trajectories demonstrates why the associations do not always represent causal relationships. Patients who experienced a relapse had an increased risk of MRI activity in either the brain or the spinal cord. This observation is biologically consistent, as clinical relapses are the symptomatic manifestation of new focal inflammatory activity. The occurrence of a clinical relapse is highly predictive of finding new disease activity on MRI (23), which leads to a confirmation through imaging. In theory, this pair should have been reversed if it were to reflect the true biological order.

Reflecting on the statistically significant difference in gender distribution of the subset in cluster b1, there is one event that could account for this observation. DMTs that fall in the OTH category, are typically identified as heavy immunosuppressive and highly effective DMTs. A recent study has shown that men were consistently escalated to these stronger therapies more quickly and frequently compared to women due to the impact of heavy immunosuppressants on family planning (24). The observed statistical difference in gender distribution can be accounted to the expected larger proportion of men switching to OTH, compared to women.

The main categories show more overarching patterns in patient progression. A logical link between EDSS increase and FSS increase is observed in the first event pair for a relatively large number of patients (7.7%). A further decrease in EDSS is linked to an increase in FSS scores, albeit with an almost negligible RR of 1.02. This last observation can be explained as the past improvement of the EDSS score of a patient, with future worsening reappearing as the disease progresses.

#### 4.1.3 5-year trajectories

The 5-year trajectories repeated many of the observations made within the 2-year trajectories. The effect of a switch to IFN/GLAT still shows an increased risk of relapse, although the increase in patient count was low. Similarly, the RRs between “*MRI BR* → *ALZ*”, “*Relapse* → *ALZ*” and “*ALZ* →−*Mental*” remain significantly elevated, with increasing values compared to the 2-year trajectories. This observation indicates that these trajectories do not get drowned out by long-term event pairs that have a connection to treatment switches to ALZ or IFN/GLAT. The small change in patient numbers further confirms that these events primarily happen within the first two years after event A rather than thereafter.

OTH is shown to improve cerebellar and brainstem FSS, while also worsening the pyramidal and ambulatory FSS. The “*OTH* → +*Ambulation*” event pair shows a high RR of 18.50. An explanation for this observation is the treatment approach applied within the tertiary MS center, where episodes of clinical worsening are managed by a short-term supplement of cyclophosphamide (OTH) to the patient’s therapeutic regimen. This further explains the appearance of “*SPMS* → *OTH*” in the trajectory table (**Table 6**) with a RR of 2.49. As OTH is often used as an additional treatment, it frequently appears in the history of patients resulting in an overrepresentation of OTH switches in the 5-year analysis. Medical experts have confirmed that OTH improved the stability of the patients’ EDSS score within the real-world MS cohort at the tertiary MS center.

Another new observations is cluster c4, which shows a high RR for “*SPMS* → +*Ambulation*” and “−*Ambulation* → +*Ambulation*” with the latter having a doubled patient count compared to the observations of the 2-year trajectories. These observations are in line with the prolonged effects of MS on patients diagnosed with progressive MS leading to an eventual worsening of their ambulation. This pattern only appears in the long term observation window as ambulatory worsening of MS after diagnosis with progressive MS is mostly gradual. A doubling in patient count signifies that these trajectories appear equally often before the two year mark after diagnosis of SPMS, but also thereafter.

Similar to OTH, results show that ALZ can lead to worsening scores such as an increase in ambulation score, but also an increase in the bowel and bladder functional system score. The negative influence of ALZ on the bowel/bladder is more difficult to explain. These treatments have known side-effects that influence the bowel/bladder system, but their clinical manifestation is infrequent and should not cause a significant difference with other treatment options (25). Furthermore, all events in **Table 6** show worsening of the FSS. These findings suggest that in the majority patients the progressive neurodegeneration cannot be fully stopped by a switch in treatment, leading to worsening of the ambulatory or bowel and bladder functional systems as seen in this MS cohort dataset.

The statistically significant difference in gender distribution on cluster c1 can be further explained with regards to the previous findings. As men are more often on heavy immunosuppressants, women are more likely to be on Interferon-beta and Glatiramer Acetate. Studies have shown that men are more likely to be escalated from IFN/GLAT to OTH (26), while women are less likely to be prescribed high efficacy therapies (24).

Lastly, within **Table 6** the improvement of one functional system is associated to the worsening of another in three pairs (“−*Mental* → +*Bowel/bladder*”, “−*Cerebellar* → +*Mental*”, “−*Pyramidal* → +*Mental*”) over a five-year time period. MS patients may compensate for deficits in one FS by overusing or straining another functional system, leading to the observed patterns above.

### 4.2 Analysis 2: the applicability of patient trajectory analysis on a small dataset

The reproduction of the Cladribine study by Aerts et al. yielded similar event and patient counts when all event pairs were considered, regardless of their RR. These findings suggest that the analytical pipeline provides correct counting of event pairs on small datasets. When looking further into the statistical findings from Aerts et al. (13), a trend of earlier disease activity for patients who received FNG prior to CLAD initiation was expected. The results have shown that these patients have in fact a statistically significant increase in RR for disease activity (EDA) within the first year of the study (*RR* = 1.33*, p <* 0.01). This aligns with the findings of Sorensen et al. (27) who observed that the rate of NEDA did not improve after changing to CLAD from FNG compared to the reduced appearance of EDA when switching from other prior DMTs. These results already indicate that the first four findings from Aerts et al. (13) are confirmed by this study.

However, when examining the effect of the last DMT used before CLAD initiation, additional statistically significant trajectories were found. FNG was associated with a significantly increased RR for relapse activity. This aligns with findings from Rauma et al. (28), who observed rebound relapses after patients switched from FNG to CLAD. This was not reported in the previous study (13). Although Zhong et al. (29) noted that a prolonged washout period before starting CLAD may elevate the risk of relapse within the first year of treatment, current literature provides little evidence that switching from TRF to CLAD is associated with increased relapse activity as observed in the “*TRF* → *Relapse*” trajectory. Furthermore, Spelman et al. (30) mention that a switch from less effective drugs such as TRF is often accompanied by stabilization of the disease when switching to CLAD, further reinforcing findings of improvement rather than worsening. Nonetheless, a possible explanation for this observation is that patients treated with TRF are more prone to relapses in general and therefore also experience relapses during CLAD treatment.

Lastly, FNG and DMF displayed an increased RR towards CDW after at least one year since CLAD initiation. These findings are difficult to attribute to previous findings in literature. However, it should also be considered that the patient count for these observations is extremely low due to the size of the dataset, only appearing in two patients. This can lead to coincidental patterns being treated as significant by the PTRA pipeline, while the small patient sample limits the ability to verify these associations through independent statistical tests.

The study of Aerts et al. (13) reported a trend toward earlier disease activity in patients who had received a higher number of prior DMTs (≥ 2 before CLAD initiation). However, our analysis revealed a distinct, statistically significant risk of EDA specifically for patients with fewer prior DMTs (*<*2 DMTs) within the first 24 months (RR = 1.20) which was consistent across all EDA subtypes (MRI, Relapse, and CDW). It is hypothesized that this discrepancy stems from two key factors: first, regarding methodology; Kaplan-Meier analyses are highly sensitive to the timing of event clusters. The “early activity” noted by (13) may have been driven by a high incidence of events specifically at the 24-month mark. In this study, while a significant risk for the “*<*2 DMTs”-group is observed in the short term (24 months), this significance was not maintained over the full study duration, aligning with the transient nature of the observation. Second, and perhaps more critical, is the impact of age-matching within the PTRA pipeline. Patients with fewer than 2 prior DMTs were significantly younger than those with ≥2 prior DMTs. Because the PTRA method matches controls by age and sex, the comparison group for the <2 DMTs cohort effectively became “young patients with ≥2 prior DMTs”. This demographic skew likely influences the RR calculations, resulting in different statistical conclusions compared to population-level studies (13, 27, 28).

### 4.3 Limitations and future research

#### 4.3.1 Limitations

When interpreting the results of these analyses, several limitations regarding the source data must be considered. First, the dataset spans a broadly extended timeline, with the earliest recorded relapse dating back to January 1969 and the dataset lock occurring in October 2024. Other early clinical milestones include the first recorded treatment change in 1979, the first MRI changes in 2003, and the earliest EDSS and FSS changes in 2008. Because the clinical understanding and diagnostic criteria for MS have evolved significantly over this long period, early historical observations might be interpreted differently by modern standards. Additionally, as the dataset is from a single MS tertiary center, local treatment regimens are portrayed in the retrieved trajectories, limiting the generalizability of the findings.

Furthermore, data limitations slightly impacted the cladribine (CLAD) reproduction analysis. Because the dataset was not strictly limited by the confirmed disability worsening records, which were a limiting factor in the study of Aerts et al. (13), it was not possible to limit the number of patients for the NEDA analysis to be identical to the previous study. However, as the overall patient counts aligned closely, the downstream impact on the results is expected to be minimal.

The analytical approach itself carries inherent constraints. Primarily, the method highlights correlations and timing-related biases within the source database but cannot establish causality. Furthermore, the effectiveness of the PTRA method heavily relies on the specific definitions of the diagnostic events. While for this study, the definitions are based on both references to previous literature and available data, it is important to acknowledge that event criteria can vary widely across academic studies, which may impact the generalizability of our findings to other cohorts.

Additionally, the PTRA method’s reliability decreases when applied to very small datasets. This was evident when testing prior DMTs for statistically significant patient trajectories as the values of the RR fluctuated between experiments, necessitating the use of mean and standard deviation. This was also notable in a subset of seven patients characterized by younger age, shorter disease duration, and fewer prior DMTs before CLAD initiation. The algorithm was forced to match these patients with similarly-ages profiles in the ≥ 2 DMT group. This led to an increased the RR for the group with fewer than two previous DMTs, while this is in stark contrast with previous findings (27, 28).

Finally, a conscious methodological trade-off was made regarding trajectory time-ranges. By setting the minimum duration between events to one year, the 5-year trajectories naturally overlapped with the 2-year trajectory results. While a strict two-year limit would have ensured mutually exclusive observations for each experiment, the one-year limit was retained to enable the comparison of statistical effects and longitudinal shifts across multiple time-ranges.

#### 4.3.2 Future work

These limitations point to several promising avenues for future research. To address the constraints of the PTRA method on small or contradictory datasets, future work should focus on developing a more developed random selection algorithm that better accounts for demographic imbalances. To move beyond correlation and establish causality, an experimental setup or comparative trial study is required. The findings that have not been confirmed by previous studies can be examined more thoroughly through a targeted trial comparing the influence of FNG or ALZ on the bowel/bladder system.

Finally, the primary strength of the PTRA method is its efficiency in assessing large longitudinal datasets. This utility could be significantly enhanced by developing interactive visualization tools that allow clinicians to rapidly inspect patient demographics within specific trajectories. Extending this approach toward predictive machine learning models would also allow researchers to forecast clinical outcomes based on these observed longitudinal patterns, ultimately providing greater prognostic value in clinical 558 practice.

## 5 Conclusion

Analysis of MS patient trajectories reveals clear patterns in disease progression that reflect both biological processes and historical treatment practices. Short-term trajectories highlight relapse-driven fluctuations and the use of alemtuzumab and older immunosuppressive agents, while also illustrating links between functional system changes, EDSS progression, and therapy switches. Long-term trajectories capture the gradual worsening of MS and uncover gender-related differences in treatment escalation, as well as previously unreported associations between ALZ and the bowel/bladder or ambulation scores. These findings underscore the value of trajectory analysis but also the need for caution given the influence of local treatment procedures.

The reproduction of the Cladribine study supports the robustness of the analytical pipeline, confirming most findings from Aerts et al. and detecting the expected increase in early disease activity after switching from fingolimod to cladribine. Additional significant trajectories, such as relapse risk after FNG or TRF, were identified, though some may reflect small sample sizes or underlying patient characteristics rather than true treatment effects. Overall, the combined analyses show that the pipeline reliably captures meaningful patterns.

## Data Availability

The data analyzed in this study is subject to the following licenses/restrictions: The dataset used is a non-public dataset. Requests to access these datasets should be directed to niels.jodts@ugent.be.
