# Supplementary material for: Data-driven hypothesis discovery from disease trajectories in multiple sclerosis
Source: Front Immunol. 2026 Apr 14;17:1758416. doi: 10.3389/fimmu.2026.1758416 (PMC13120929; doi:10.3389/fimmu.2026.1758416)
Supplement: Supplementary file 1 [file DataSheet1.pdf]

1 SUPPLEMENTARY MATIERIAL

Table 1. All 1-year trajectories, sorted by relative risk (RR) score. These trajectory show a statistically significant increase in RR score ($p < 0.01$). Absolute and relative patient count are shown (n=985).

Cluster	Patients (%)	RR	Cluster	Patients (%)	RR
OTH → - Ambulation	14 (1.42%)	7.00	MRI BR → MRI SC	12 (1.22%)	1.20
+ Mental → - Mental	19 (1.93%)	2.38	SPMS → OTH	40 (4.06%)	1.18
+FSS → ALZ	14 (1.42%)	1.75	Relapse → ALZ	19 (1.93%)	1.12
+ Ambulation → OTH	25 (2.54%)	1.25	ALZ → -EDSS	27 (2.74%)	1.04

Table 2. All 2-year trajectories, sorted by relative risk (RR) score. These trajectory show a statistically significant increase in RR score ($p < 0.01$). Absolute and relative patient count are shown (n=985).

Cluster	Patients (%)	RR	Cluster	Patients (%)	RR
CDW → + Ambulation	17 (1.73%)	17.00	IFN/GLAT → Relapse	144 (14.62%)	1.36
- Ambulation → + Ambulation	10 (1.02%)	5.00	MRI BR → ALZ	24 (2.44%)	1.33
+ Mental → - Mental	33 (3.35%)	4.71	-FSS → + Mental	10 (1.02%)	1.25
+FSS → + Ambulation	10 (1.02%)	3.33	CDW → - Sensory	11 (1.12%)	1.22
CDW → +FSS	17 (1.73%)	2.83	OTH → -FSS	17 (1.73%)	1.21
+ Mental → + Ambulation	12 (1.22%)	2.40	Relapse → MRI SC	18 (1.83%)	1.20
CDW → + Pyramidal	19 (1.93%)	2.38	CDI → + Ambulation	13 (1.32%)	1.18
+FSS → ALZ	16 (1.62%)	2.00	Relapse → MRI BR	44 (4.47%)	1.13
+ Pyramidal → - Pyramidal	10 (1.02%)	2.00	FNG → ALZ	18 (1.83%)	1.12
CDW → + Bowel/Bladder	15 (1.52%)	1.88	+ Pyramidal → ALZ	10 (1.02%)	1.11
CDW → ALZ	33 (3.35%)	1.74	Relapse → + Sensory	10 (1.02%)	1.11
+ Ambulation → OTH	29 (2.94%)	1.71	CDW → CDI	68 (6.90%)	1.10
FNG → + Bowel/Bladder	10 (1.02%)	1.67	OTH → + Bowel/Bladder	11 (1.12%)	1.10
SPMS → OTH	59 (5.99%)	1.59	Relapse → -FSS	11 (1.12%)	1.10
Relapse → ALZ	27 (2.74%)	1.59	CDW → OTH	78 (7.92%)	1.08
+FSS → - Mental	11 (1.12%)	1.57	OTH → CDI	69 (7.01%)	1.05
ALZ → - Mental	13 (1.32%)	1.44	FNG → + Mental	20 (2.03%)	1.05

Table 3. All 5-year trajectories, sorted by relative risk (RR). These trajectory show a statistically significant increase in RR score ($p < 0.01$). Absolute and relative patient count are shown (n=985).

Cluster	Patients (%)	RR	Cluster	Patients (%)	RR
OTH → + Ambulation	37 (3.76%)	18.50	-FSS → + Mental	10 (1.02%)	1.43
+FSS → + Ambulation	22 (2.23%)	7.33	Relapse → - Mental	28 (2.84%)	1.40
- Ambulation → + Ambulation	20 (2.03%)	6.67	IFN/GLAT → + Bowel/Bladder	14 (1.42%)	1.40
SPMS → - Ambulation	16 (1.62%)	4.00	CDW → + Bowel/Bladder	14 (1.42%)	1.40
+ Mental → + Ambulation	18 (1.83%)	3.00	MRI BR → ALZ	25 (2.54%)	1.39
+ Mental → + Pyramidal	20 (2.03%)	2.86	FNG → + Bowel/Bladder	11 (1.12%)	1.38
ALZ → + Bowel/Bladder	17 (1.73%)	2.83	OTH → + Pyramidal	18 (1.83%)	1.29
OTH → - Cerebellar	16 (1.62%)	2.67	CDW → + Sensory	10 (1.02%)	1.25
ALZ → + Ambulation	16 (1.62%)	2.67	OTH → - BrainStem	10 (1.02%)	1.25
SPMS → OTH	87 (8.83%)	2.49	Relapse → FNG	42 (4.26%)	1.24
SPMS → OTH	87 (8.83%)	2.49	SPMS → ALZ	21 (2.13%)	1.24
+ Mental → + Sensory	12 (1.22%)	2.40	FNG → - Mental	21 (2.13%)	1.24
SPMS → + Ambulation	12 (1.22%)	2.40	Relapse → + Mental	28 (2.84%)	1.22
CDW → - Cerebellar	12 (1.22%)	2.40	CDI → CDW	98 (9.95%)	1.21
-FSS → ALZ	14 (1.42%)	2.33	CDI → -FSS	12 (1.22%)	1.20
OTH → +FSS	29 (2.94%)	2.23	OTH → -FSS	19 (1.93%)	1.19
CDI → + Ambulation	29 (2.94%)	2.23	NTZ → ALZ	27 (2.74%)	1.17
Relapse → ALZ	29 (2.94%)	2.23	FNG → + Mental	25 (2.54%)	1.14
+FSS → + Mental	20 (2.03%)	2.22	Relapse → + Pyramidal	16 (1.62%)	1.14
- Cerebellar → + Mental	11 (1.12%)	2.20	DMF → CLA	54 (5.48%)	1.12
Relapse → + Sensory	15 (1.52%)	2.14	MRI BR → - Mental	21 (2.13%)	1.11
Relapse → + Bowel/Bladder	15 (1.52%)	2.14	FNG → - Pyramidal	10 (1.02%)	1.11
ALZ → - Mental	22 (2.23%)	2.00	CDI → - Cerebellar	10 (1.02%)	1.11
- Pyramidal → + Mental	12 (1.22%)	2.00	OTH → + Bowel/Bladder	11 (1.12%)	1.10
Relapse → - Cerebellar	12 (1.22%)	2.00	IFN/GLAT → NTZ	80 (8.12%)	1.07
- Pyramidal → ALZ	11 (1.12%)	1.83	IFN/GLAT → NTZ	80 (8.12%)	1.07
FNG → ALZ	30 (3.05%)	1.67	Relapse → +FSS	19 (1.93%)	1.06
CDW → + Pyramidal	15 (1.52%)	1.67	Relapse → MRI SC	18 (1.83%)	1.06
- Mental → + Bowel/Bladder	10 (1.02%)	1.67	OTH → CDI	62 (6.29%)	1.05
SPMS → +FSS	17 (1.73%)	1.55	OTH → CDI	62 (6.29%)	1.05
IFN/GLAT → Relapse	152 (15.43%)	1.49	NTZ → FNG	39 (3.96%)	1.03
IFN/GLAT → Relapse	152 (15.43%)	1.49	Relapse → MRI BR	48 (4.87%)	1.02
Relapse → - Pyramidal	13 (1.32%)	1.44	Relapse → MRI BR	48 (4.87%)	1.02
